# Trends and disparities in breast cancer mortality among older adults in the United States, 1999–2020

**DOI:** 10.3389/fonc.2026.1914451

**Published:** 2026-09-25

**Authors:** Tong Feng, Pengcheng Zheng, Qingyuan Li, Ding Han, Ran Duan

**Affiliations:** 1 Department of Respiratory and Critical Care Medicine, Deyang People’s Hospital, Deyang, China; 2 School of Clinical Medicine, Chengdu Medical College, Chengdu, China; 3 Department of Respiratory and Critical Care Medicine, The First Affiliated Hospital of Chengdu Medical College, Chengdu, China; 4 Laboratory of Geriatric Respiratory Diseases of Sichuan Higher Education Institute, Chengdu, China; 5 Department of Oncology, The First Affiliated Hospital of Chengdu Medical College, Chengdu, China

**Keywords:** breast cancer, CDC WONDER, health disparities, Joinpoint regression, mortality, older adults, United States

## Abstract

**Background:**

Breast cancer remains a major cause of cancer mortality among older adults in the United States. Although overall breast cancer mortality has declined in recent decades, the extent to which these improvements have been shared across demographic and geographic subgroups among adults aged 65 years and older remains insufficiently characterized.

**Methods:**

We conducted a population-based repeated cross-sectional mortality analysis using the CDC WONDER Underlying Cause of Death database from 1999 to 2020. Deaths due to breast cancer among U.S. adults aged 65 years and older were defined using ICD-10 code C50 as the underlying cause of death. Age-adjusted mortality rates (AAMRs) and age-specific crude mortality rates were calculated. Female-specific AAMRs were used as the primary outcome for demographic, geographic, state-level SDI panel, and descriptive frontier analyses; male breast cancer mortality was reported separately as a descriptive finding. Temporal trends were assessed using Joinpoint regression.

**Results:**

From 1999 to 2020, 544,812 deaths due to breast cancer occurred among U.S. adults aged 65 years and older, including 538,489 deaths among women and 6,323 among men. The pooled female-specific AAMR was 99.84 per 100,000 women, compared with 1.70 per 100,000 men. Among older women, mortality increased with age, was highest among Black or African American women, and varied substantially across regions and jurisdictions. The combined-sex AAMR declined from 71.58 to 50.83 per 100,000 population, although the decline slowed after 2014. State-level analyses showed marked geographic and sociodemographic variation in female breast cancer mortality.

**Conclusions:**

Breast cancer mortality among older U.S. adults declined significantly between 1999 and 2020, but the increasing absolute number of deaths and persistent age, racial, ethnic, and geographic disparities indicate a continuing public health burden. Targeted strategies to improve equitable access to screening, timely diagnosis, guideline-concordant treatment, and geriatric oncology care are needed to further reduce mortality among older adults.

## Graphical Abstract

**Figure:**
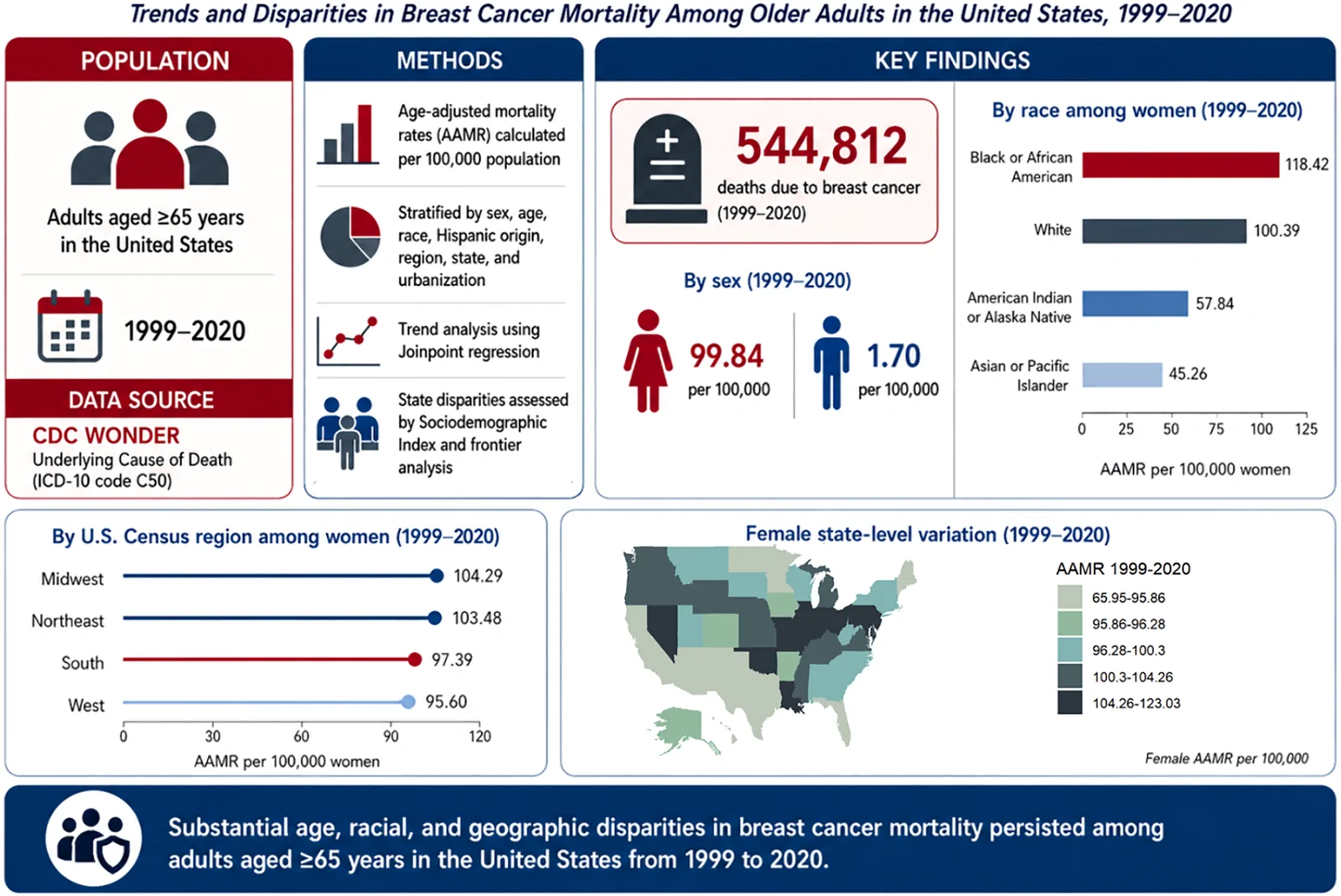

## Introduction

Breast cancer remains one of the most important public health challenges worldwide and continues to be among the leading causes of cancer morbidity and mortality in women (1). In the United States, breast cancer represents the most commonly diagnosed cancer among women and accounts for a substantial proportion of cancer-related deaths despite major advances in screening, diagnosis, and treatment (2). Over recent decades, population-based studies have demonstrated a marked decline in breast cancer mortality, largely attributable to the combined effects of mammographic screening, earlier detection, improved locoregional management, and advances in systemic therapy (3). However, the benefits of these advances have not been distributed equally across all age groups, racial and ethnic populations, and geographic settings.

Older adults constitute a particularly vulnerable and increasingly important population in breast cancer control. With population aging, the absolute burden of breast cancer among individuals aged ≥65 years is expected to rise, and older patients frequently present with distinctive clinical and public health challenges (4, 5). Compared with younger patients, older adults with breast cancer often differ substantially in screening patterns, comorbidity burden, functional status, frailty, competing mortality risks, treatment tolerance, and preferences for care. These factors may influence decisions regarding mammographic screening, diagnostic evaluation, surgery, radiotherapy, endocrine therapy, chemotherapy selection, and eligibility for HER2-targeted or other systemic therapies. Although some older patients have biologically favorable tumor characteristics, they may be diagnosed at more advanced stages and are more likely to receive less intensive or non-guideline-concordant treatment than younger patients (6). Contemporary geriatric breast oncology therefore emphasizes individualized decision-making based not on chronological age alone, but on life expectancy, comorbidities, functional and cognitive status, frailty, social support, treatment goals, and geriatric-assessment findings. National surveillance studies further indicate that declines in breast cancer mortality vary by race/ethnicity, age group, and U.S. Census region, with smaller mortality reductions observed among women aged 65 years or older and among women living in the South (7). These considerations highlight the need for population-level analyses focused specifically on breast cancer mortality among older adults.

Although prior national analyses have described breast cancer mortality trends by age, race/ethnicity, and geographic region in the general population (8–11), several important gaps remain for older adults. First, relatively few studies have focused specifically on adults aged ≥65 years, who differ from younger patients in screening patterns, comorbidity burden, competing mortality risks, frailty, treatment tolerance, treatment selection, and geriatric-assessment needs. Second, state-level variation in breast cancer mortality among older adults has not been fully characterized in conjunction with demographic and regional disparities. Third, the relationship between state-level sociodemographic development and mortality burden has not been systematically evaluated using the Socio-Demographic Index and frontier-based benchmarking approaches. Therefore, the novelty of this study lies in its integrated focus on adults aged 65 years and older, comprehensive assessment of demographic and state-level disparities, and incorporation of state-level SDI and frontier-based benchmarking analyses.

Therefore, this study aimed to evaluate long-term trends and disparities in breast cancer mortality among adults aged 65 years and older in the United States from 1999 to 2020. We assessed mortality patterns by sex, age group, race, Hispanic origin, U.S. Census region, state of residence, and urbanization status; examined temporal changes using Joinpoint regression; and evaluated the relationship between state-level sociodemographic development and female breast cancer mortality. Female-specific AAMRs were treated as the primary outcome, while male breast cancer mortality was reported separately as a descriptive surveillance finding.

## Methods

### Study design and data source

We conducted a population-based repeated cross-sectional mortality analysis using aggregate death-certificate data from the CDC WONDER Underlying Cause of Death database (12). This database contains mortality records derived from death certificates for U.S. residents and corresponding population estimates. Individual-level follow-up, exposure assessment, and clinical cohort construction were not performed.

The study period extended from January 1, 1999, to December 31, 2020. This period was selected because the CDC WONDER Underlying Cause of Death file based on bridged-race categories provides nationally comparable ICD-10-coded mortality and population data for 1999–2020. Using this file allowed consistent classification of race, Hispanic origin, population denominators, geographic variables, and urbanization status across the entire study period. Although more recent mortality data are available in CDC WONDER through the single-race mortality file, those data use a different race classification framework and constitute a separate data series; therefore, they were not appended to the present analysis to avoid potential discontinuity in long-term race- and ethnicity-specific trend estimates.

Because this study used publicly available, deidentified, aggregate mortality data, institutional review board approval and informed consent were not required.

### Study population and ascertainment of deaths due to breast cancer

The study population comprised adults aged 65 years and older residing in the United States. Deaths due to breast cancer were identified when breast cancer was recorded as the underlying cause of death on the death certificate. The underlying cause of death was defined as the disease or condition that initiated the sequence of events leading directly to death. Deaths in which breast cancer was recorded only as a contributing or associated condition were not included. Breast cancer was identified using the International Classification of Diseases, Tenth Revision (ICD-10) code C50.

### Demographic and geographic variables

Mortality data were extracted by calendar year and stratified according to sex, age group, race, Hispanic origin, U.S. Census region, state of residence, and urbanization status.

Age-specific analyses were conducted for adults aged 65–74 years, 75–84 years, and 85 years or older. Sex was categorized as female or male. Race was categorized as White, Black or African American, American Indian or Alaska Native, and Asian or Pacific Islander. Hispanic origin was analyzed separately as Hispanic or Latino and non-Hispanic or non-Latino. U.S. Census regions were categorized as Northeast, Midwest, South, and West. Urbanization status was classified as metropolitan or nonmetropolitan according to the categories available in CDC WONDER.

### Mortality-rate estimation

The numbers of deaths, annual population denominators, crude mortality rates, age-specific crude mortality rates, and age-adjusted mortality rates (AAMRs) were obtained from CDC WONDER. Mortality rates were expressed as deaths per 100,000 population. Age-specific rates were crude rates calculated within each age stratum and were not age-adjusted.

For calendar-year-specific analyses, the denominator represented the corresponding annual resident population estimate. For estimates pooled across 1999–2020, the denominator represented the cumulative population denominator, calculated as the sum of annual population estimates across all years in the study period and relevant strata. Thus, pooled denominators should be interpreted as person-year-like denominators rather than counts of unique individuals.

AAMRs were calculated using the direct standardization method and the 2000 U.S. standard population. Because the study population was restricted to adults aged 65 years and older, age adjustment was performed within the ≥65-year population rather than across the entire age range. Specifically, age-specific mortality rates for three older-adult age strata—65–74 years, 75–84 years, and 85 years or older—were weighted according to the corresponding 2000 U.S. standard population distribution, with weights normalized to sum to 1 across these three age groups. The resulting AAMRs therefore represent age-adjusted mortality rates for the older-adult population and were used for comparisons across calendar years and demographic or geographic subgroups.

In accordance with CDC WONDER confidentiality requirements, estimates based on fewer than 10 deaths were suppressed. Rates based on fewer than 20 deaths were considered statistically unreliable and were not interpreted as stable estimates.

### Temporal trend analysis

Temporal trends in annual AAMRs were evaluated using the Joinpoint Regression Program, version 5.4.0, released in April 2025 by the Surveillance Research Program of the National Cancer Institute (13, 14).

Piecewise log-linear regression models were fitted to annual AAMRs to identify inflection points at which the direction or magnitude of mortality trends changed. Annual percent changes (APCs) and corresponding 95% confidence intervals (CIs) were calculated for each trend segment. Average annual percent changes (AAPCs) and corresponding 95% CIs were calculated to summarize the overall trends during the study period.

Using the default settings of Joinpoint version 5.4.0, the optimal number of joinpoints was selected using the weighted Bayesian information criterion, and confidence intervals were calculated using the empirical quantile method. Statistical significance was assessed using two-sided tests, with a P value of less than 0.05 considered statistically significant.

Joinpoint regression analyses were conducted for the overall older-adult population and for prespecified strata with stable and interpretable annual AAMRs, including sex, race, U.S. Census region, and urbanization status. Age group–specific mortality rates and Hispanic-origin-specific mortality estimates were summarized descriptively. State-level analyses were used to evaluate geographic variation and sociodemographic benchmarking rather than to estimate state-specific Joinpoint trends. Accordingly, state-specific Joinpoint regression models were not generated, and no state-specific temporal trend claims were made.

### Sociodemographic index, state-level panel, and frontier analyses

State-level mortality data were obtained from the CDC WONDER Underlying Cause of Death database for each calendar year from 1999 to 2020. For the primary state-level analyses, deaths due to breast cancer among women aged 65 years and older and the corresponding female population denominators were extracted by jurisdiction and year, and female-specific AAMRs were calculated per 100,000 women using the 2000 U.S. standard population. Male state-level analyses were not conducted because sparse death counts after stratification produced unstable, suppressed, or unavailable estimates. The resulting female state-year dataset was used for the sociodemographic and frontier analyses.

Annual state-specific Socio-Demographic Index (SDI) estimates were obtained from the Global Burden of Disease Study 2021 Socio-Demographic Index 1950–2021 dataset, produced by the Institute for Health Metrics and Evaluation. We extracted SDI estimates for the 50 U.S. states and the District of Columbia for each calendar year from 1999 through 2020 and linked them to the state-level mortality data using jurisdiction and calendar year (15).

SDI is a composite indicator of sociodemographic development calculated as the geometric mean of three normalized indices: lag-distributed income per capita, mean educational attainment among persons aged 15 years or older, and total fertility among persons younger than 25 years. SDI ranges theoretically from 0 to 1, with higher values indicating higher sociodemographic development. The published annual point estimates were used directly, and no additional sample-specific min–max normalization, z-score standardization, logarithmic transformation, or categorization was applied. SDI was therefore treated as a state-specific and time-varying continuous variable. For the panel regression, SDI was divided by 0.10 so that the regression coefficient represented the percentage change in AAMR associated with a 0.10-unit increase in SDI.

### State-level SDI panel analysis

To account for repeated annual observations within jurisdictions and temporal changes shared across jurisdictions, the association between state-level SDI and female breast cancer AAMR was evaluated using a two-way fixed-effects panel regression model. The analytic dataset included 1,118 jurisdiction-year observations from the 50 U.S. states and the District of Columbia during 1999–2020. Four observations from Alaska were excluded because the corresponding AAMRs were unavailable. For jurisdiction i in calendar year t, the model was specified as:


$$log(AAMR_{it})=\beta \;SDI_{it}+\alpha_{i}+\lambda_{t}+\epsilon_{it}$$

Jurisdiction fixed effects controlled for measured and unmeasured time-invariant differences between jurisdictions, whereas calendar-year fixed effects accounted for common national temporal trends and year-specific factors shared across jurisdictions. Cluster-robust standard errors were calculated at the jurisdiction level to account for serial correlation and heteroscedasticity associated with repeated observations. The natural logarithm of AAMR was used as the dependent variable, and SDI was scaled in 0.10-unit increments. The primary model was weighted by the inverse variance of the log-transformed AAMR. An unweighted two-way fixed-effects model was conducted as a sensitivity analysis. Regression coefficients were exponentiated and expressed as percentage changes in AAMR.

### Frontier analysis

Frontier analysis was conducted using the 2020 cross-sectional data. The analysis included 50 jurisdictions for which both the female-specific 2020 AAMR and SDI were available. Restricting the analysis to a common calendar year allowed jurisdictions to be evaluated within the same national temporal context.

A two-stage descriptive frontier procedure was used to characterize the nonlinear relationship between the Socio-Demographic Index and AAMR. First, the free-disposal principle was used to characterize the empirical lower boundary of AAMR across the observed SDI distribution. Second, LOESS regression was applied to the boundary estimates using a prespecified smoothing span of 0.30 to generate a continuous descriptive frontier curve. The LOESS curve reduced the irregularity of the empirical boundary and summarized the nonlinear pattern between SDI and AAMR; it should not be interpreted as a conventional step-function free-disposal hull estimator or as a causal or theoretical minimum mortality function.

To reduce the dependence of the displayed smooth curve on a single resampled dataset, 1,000 jurisdiction-level bootstrap samples were generated. Within each resample, the free-disposal-based boundary and LOESS curve were re-estimated, and the frontier AAMR values were evaluated at the original observed SDI values. The final smoothed frontier AAMR at each SDI value was calculated as the mean of the corresponding frontier values across the 1,000 resamples.

For jurisdiction i, the smoothed frontier gap was calculated as:


$${\text{Smoothed frontier gap}}_{\text{i}}={\text{Observed AAMR}}_{\text{i}}-{\text{Smoothed frontier AAMR}}_{\text{i}}$$

Gaps were expressed as deaths per 100,000 women aged 65 years or older. A smaller gap indicated that the observed AAMR was closer to the estimated smooth lower frontier, whereas a larger gap indicated a greater descriptive difference from that curve. The gaps were interpreted as descriptive comparative measures and not as estimates of preventable deaths, achievable mortality reductions, causal effects, or formal measures of efficiency.

### Bootstrap procedure

The bootstrap procedure was used only to construct an ensemble-averaged descriptive curve and was not used for statistical inference. Because an ordinary nonparametric bootstrap may not provide valid inferential confidence intervals for free-disposal-type boundary estimators, no bootstrap confidence intervals, P values, hypothesis tests, or ranking-stability probabilities were calculated for the smoothed frontier estimates or gaps. The frontier analysis was therefore interpreted exclusively as descriptive.

Because the panel and frontier analyses were ecological and based on aggregate jurisdiction-level data, their results were interpreted as descriptive jurisdiction-level associations and comparative measures rather than evidence of individual-level or causal effects. These analyses cannot identify the specific clinical, social, or health-system mechanisms responsible for geographic mortality patterns.

### Statistical analysis

Joinpoint regression analyses were performed using the Joinpoint Regression Program, version 5.4.0. State-level panel regression, SDI analyses, descriptive frontier analyses, bootstrap curve construction, and data visualization were conducted using R software, version 4.5.1. All inferential statistical tests were two-sided, and a P value of less than 0.05 was considered statistically significant.

## Results

### Overall mortality burden and demographic disparities

From 1999 to 2020, 544,812 deaths due to breast cancer occurred among U.S. adults aged 65 years and older, including 538,489 deaths among women and 6,323 deaths among men. Consistent with the underlying sex-specific epidemiology of breast cancer, mortality was overwhelmingly concentrated among women. Therefore, female-specific age-adjusted mortality rates (AAMRs) were used as the primary clinically interpretable estimates. Among older women, the overall AAMR was 99.84 per 100,000 women, whereas the male-specific AAMR was 1.70 per 100,000 men and was reported separately for completeness rather than interpreted as a sex-based disparity. The combined-sex AAMR of 59.03 per 100,000 older adults was retained only as a secondary population-level estimate because it includes men in the denominator, among whom breast cancer mortality was rare.

Substantial variation in female-specific AAMRs was observed across demographic and geographic subgroups. Regionally, female-specific AAMRs were highest in the Midwest and Northeast and lower in the South and West. By Hispanic origin, non-Hispanic older women had a higher AAMR than Hispanic older women. By race, Black or African American older women had the highest AAMR, whereas Asian or Pacific Islander older women had the lowest. Metropolitan and nonmetropolitan areas showed broadly comparable female-specific AAMRs. Thus, subsequent disparity interpretation focused on differences by race, Hispanic origin, U.S. Census region, state of residence, and urbanization status among older women (**Table 1**).

**Table 1:** Breast cancer mortality among adults aged ≥65 years in the United States, 1999–2020.

Group	Deaths/cumulative population denominator	AAMR per 100,000 (95% CI)
Female (primary analysis)	538,489/527,632,126	99.84 (99.57–100.10)
Census region		
Midwest	128,036/118,322,431	104.29 (103.72–104.87)
Northeast	112,801/104,310,001	103.48 (102.87–104.09)
South	191,101/194,863,980	97.39 (96.95–97.83)
West	106,551/110,135,714	95.60 (95.02–96.17)
Hispanic origin		
Hispanic or Latino	22,349/37,233,309	61.64 (60.83–62.46)
Not Hispanic or Latino	515,118/490,398,817	102.46 (102.17–102.74)
Race		
American Indian or Alaska Native	1,843/3,366,322	57.84 (55.15–60.52)
Asian or Pacific Islander	9,023/20,410,413	45.26 (44.32–46.20)
Black or African American	58,924/50,364,189	118.42 (117.46–119.38)
White	468,699/453,491,202	100.39 (100.11–100.68)
Urbanization		
Metropolitan	444,040/434,206,015	100.12 (99.83–100.42)
Nonmetropolitan	94,449/93,425,411	98.68 (98.05–99.32)
Male (descriptive)	6,323/400,844,539	1.70 (1.66–1.74)
Combined sex (secondary)	544,812/928,476,665	59.03 (58.88–59.19)

Deaths with unknown or missing Hispanic origin were included in the female and combined-sex totals but were not included in Hispanic-origin-specific subgroup comparisons. Female-specific AAMRs are the primary clinically interpretable estimates; male and combined-sex estimates are presented descriptively. AAMR, age-adjusted mortality rate; CI, confidence interval.

In the primary female state-level analysis, mortality varied markedly across jurisdictions, ranging from 65.95 per 100,000 women in Hawaii to 123.03 per 100,000 women in the District of Columbia. The lowest female-specific AAMRs were observed in Hawaii, Florida, Arizona, New Mexico, and Maine. Female-specific and combined-sex state estimates for all 50 U.S. states and the District of Columbia are reported in **Supplementary Table 1**; the combined-sex estimates are presented as secondary descriptive results (**Figure 1**; **Supplementary Table 1**).

**Figure 1:**
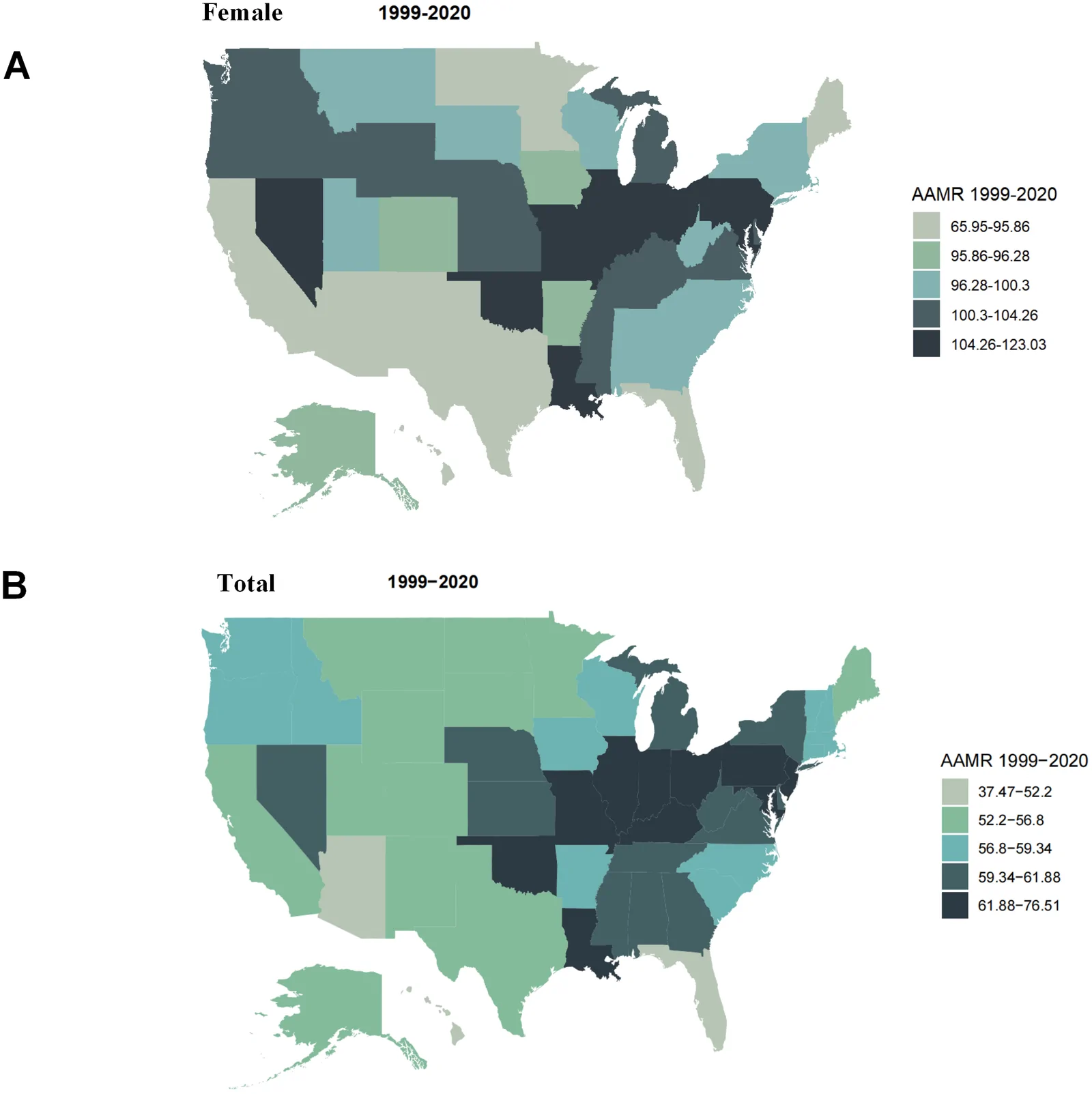
State-level age-adjusted breast cancer mortality rates among adults aged ≥65 years in the United States and the District of Columbia, 1999–2020. **(A)** Female-specific AAMRs per 100,000 women (primary analysis); **(B)** combined-sex AAMRs per 100,000 adults (secondary descriptive analysis). AAMR, age-adjusted mortality rate.

### Age-specific mortality patterns

Age-specific crude mortality rates increased with age in both 1999 and 2020. In 2020, the age-specific crude mortality rates were 33.03, 55.89, and 112.10 per 100,000 population among adults aged 65–74, 75–84, and 85 years or older, respectively. These age-stratum-specific estimates were not age-adjusted. Consistent with the underlying sex-specific epidemiology of breast cancer, age-specific crude mortality rates were higher among women than men across all age groups, with the highest rates observed among women aged 85 years or older (**Supplementary Table 2**; **Supplementary Figure 1**). Age- and sex-specific trend plots further showed persistent age gradients and overall declines across most strata during the study period (**Supplementary Figure 2**).

### Temporal trends in age-adjusted mortality rates

The overall AAMR declined from 71.58 per 100,000 population in 1999 to 50.83 per 100,000 population in 2020, despite an increase in the annual number of deaths from 24,760 to 27,409 (**Supplementary Table 3**; **Supplementary Figure 3**). Joinpoint analysis showed a statistically significant decline from 1999 to 2014 (APC, –1.871%; P < 0.001), followed by an apparent slowing of the decline from 2014 to 2020; however, this later segment did not reach statistical significance (APC, –1.061%; P = 0.074). The overall AAPC was –1.64% (P < 0.001) (**Table 2**; **Figure 2A**).

**Table 2:** Joinpoint regression analysis of trends in breast cancer rates by demographic and geographic characteristics, United States, 1999–2020.

Variable	Segment	Segment start	Segment end	APC, % (95% CI)	P value	AAPC, % (95% CI)	P value
Total	1	1999	2014	-1.871(-2.187, -1.756)	<0.001	-1.64(-1.751, -1.552)	<0.001
	2	2014	2020	-1.061(-1.523, 0.201)	0.074		
Sex							
Female	1	1999	2013	-1.547(-2.045, -1.429)	0.004	-1.36(-1.462, -1.274)	<0.001
	2	2013	2020	-0.985(-1.306, 0.063)	0.056		
Male	1	1999	2020	-1.163(-1.597, -0.658)	<0.001	-1.163(-1.597, -0.658)	<0.001
Race							
American Indian or Alaska Native	1	1999	2020	-1.346(-2.465, 0.085)	0.063	-1.346(-2.465, 0.085)	0.063
Asian or Pacific Islander	1	1999	2020	0.245(-0.159, 0.761)	0.193	0.245(-0.159, 0.761)	0.193
Black or African American	1	1999	2020	-1.228(-1.406, -1.027)	<0.001	-1.228(-1.406, -1.027)	<0.001
White	1	1999	2013	-1.93(-2.146, -1.812)	<0.001	-1.672(-1.758, -1.589)	<0.001
	2	2013	2020	-1.156(-1.474, -0.35)	0.022		
Urbanization							
Metro	1	1999	2013	-1.976(-2.464, -1.837)	<0.001	-1.729(-1.848, -1.622)	<0.001
	2	2013	2020	-1.234(-1.632, -0.056)	0.045		
Nonmetro	1	1999	2018	-1.438(-2.027, -1.162)	0.027	-1.211(-1.432, -1.088)	<0.001
	2	2018	2020	0.973(-1.369, 2.347)	0.615		
Census Region							
Northeast	1	1999	2014	-2.441(-3.453, -2.236)	0.005	-2.223(-2.397, -2.082)	<0.001
	2	2014	2020	-1.675(-2.287, 0.181)	0.067		
Midwest	1	1999	2004	-2.489(-4.016, -1.842)	<0.001	-1.856(-1.964, -1.709)	<0.001
	2	2004	2020	-1.657(-1.791, -1.031)	0.006		
South	1	1999	2013	-1.558(-3.075, 0.547)	0.059	-1.333(-1.537, -1.138)	<0.001
	2	2013	2020	-0.881(-1.521, 1.047)	0.238		
West	1	1999	2014	-1.588(-2.433, -1.399)	0.02	-1.366(-1.518, -1.237)	<0.001
	2	2014	2020	-0.809(-1.373, 0.91)	0.223		

**Figure 2:**
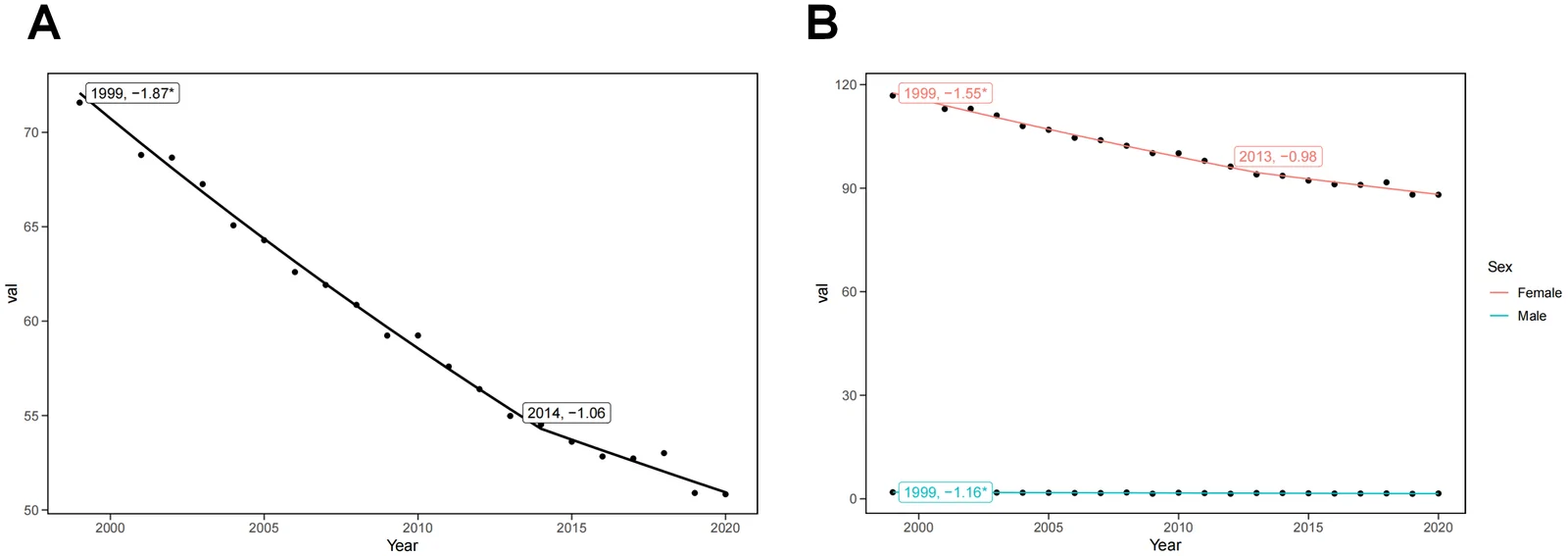
Joinpoint regression analysis of breast cancer age-adjusted mortality rate trends among adults aged ≥65 years, 1999–2020. **(A)** Combined-sex trend; **(B)** sex-specific trends. APC, annual percent change.

Sex-specific analyses showed significant overall declines in both women and men. Among women, mortality declined significantly from 1999 to 2013 and then showed an apparent slower decline after 2013. Among men, mortality declined steadily throughout 1999–2020 (**Figure 2B**; **Supplementary Table 4**). Declining trends were also observed across most racial, urbanization, and regional subgroups, although the magnitude and statistical significance varied by subgroup (**Table 2**).

### State-level SDI and frontier analyses

In the pooled jurisdiction-year data, SDI and AAMR showed a modest inverse rank correlation **Supplementary Figure 4**.

The final 2020 frontier analysis included 50 jurisdictions with available AAMR and SDI data. The five jurisdictions with the smallest smoothed frontier gaps were Hawaii (17.24 deaths per 100,000), North Dakota (19.62), Rhode Island (26.42), Florida (27.20), and Idaho (29.10). The five jurisdictions with the largest smoothed frontier gaps were Wyoming (62.62), Nebraska (55.18), South Dakota (52.23), Kentucky (52.19), and Indiana (52.07), in descending order. The observed AAMR, SDI, LOESS-smoothed frontier AAMR, smoothed frontier gap, direction of AAMR change from 1999 to 2020, and gap rank for each jurisdiction are reported in **Supplementary Table 5**. These gaps represent descriptive vertical differences between the observed 2020 AAMRs and the estimated smooth frontier and should not be interpreted as estimates of preventable mortality or causal intervention effects (**Figure 3**).

**Figure 3:**
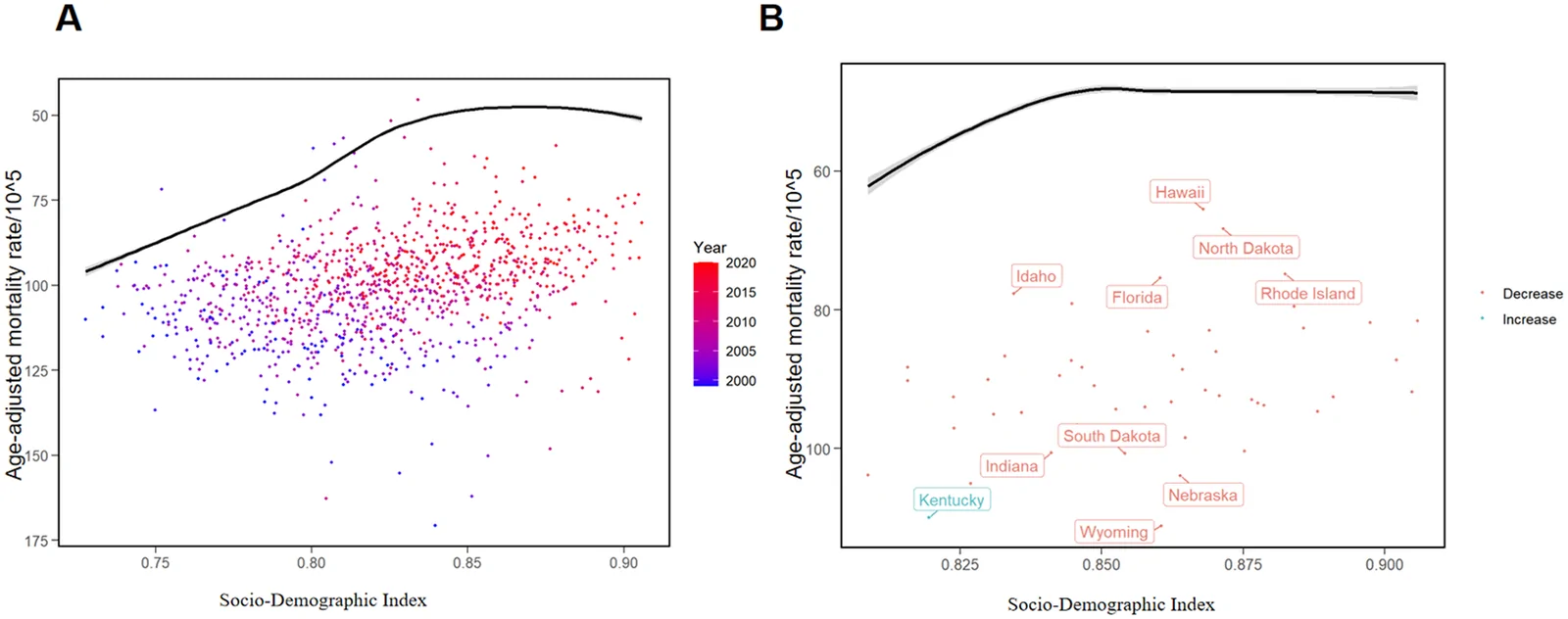
Descriptive analysis of the relationship between the Socio-Demographic Index and female-specific breast cancer age-adjusted mortality rates among women aged ≥65 years. **(A)** Jurisdiction-level observations from 1999 to 2020 showing the relationship between the Socio-Demographic Index and AAMR. **(B)** Descriptive frontier analysis based on the 2020 cross-sectional data. The solid curve represents the LOESS-smoothed lower frontier derived from the free-disposal-based boundary estimates, using a smoothing span of 0.30 and averaging the frontier AAMR values across 1,000 jurisdiction-level bootstrap resamples. The vertical difference between each observed AAMR and the corresponding smooth curve represents the smoothed frontier gap. Orange points indicate jurisdictions in which AAMR decreased from 1999 to 2020, and green points indicate jurisdictions in which AAMR increased during this period. Labels identify the five jurisdictions with the smallest and five jurisdictions with the largest smoothed frontier gaps. The analysis is descriptive, and no inferential uncertainty intervals are presented. AAMR, age-adjusted mortality rate; SDI, Socio-Demographic Index.

## Discussion

In this population-based repeated cross-sectional mortality analysis of U.S. adults aged 65 years and older, breast cancer mortality declined substantially from 1999 to 2020, with an overall AAPC of −1.64%. This pattern is consistent with the broader national decline in breast cancer mortality observed over recent decades (2). Nevertheless, the annual absolute number of deaths due to breast cancer increased during the study period, underscoring the growing demographic impact of population aging. Prior studies indicate that the cancer burden is increasingly concentrated among older adults and that cancer survivorship among older individuals is expanding substantially in the United States (16, 17). Thus, although age-adjusted mortality rates have decreased, the absolute burden of breast cancer mortality among older adults remains substantial and continues to pose challenges for oncology, geriatric medicine, and public health systems.

A notable finding of this study was the apparent deceleration in the decline in mortality after approximately 2013–2014. Similar slowing of progress has been reported in national analyses stratified by age, race/ethnicity, and U.S. Census region, particularly among women aged 65 years or older (7). However, the reasons for this pattern cannot be determined from CDC WONDER data alone. Because this database contains aggregate mortality information and lacks individual-level data on screening history, stage at diagnosis, tumor subtype, treatment receipt, comorbidities, frailty, socioeconomic status, and health-care access, the observed deceleration should be interpreted as a descriptive trend rather than evidence of specific causal mechanisms. Although the study period included the first year of the COVID-19 pandemic, data through 2020 are insufficient to evaluate the downstream effects of pandemic-related diagnostic delays or treatment disruptions on breast cancer mortality among older adults. Prior studies have documented pandemic-related reductions or delays in cancer screening, diagnosis, and treatment, and simulation modeling suggests that such disruptions may have a measurable but uncertain long-term impact on breast cancer mortality (18, 19). Longer follow-up and linked clinical data will be needed to assess whether the pandemic had any measurable impact on subsequent mortality patterns.

Racial differences in breast cancer mortality should also be interpreted with caution. In this study, Black or African American older women had the highest female-specific age-adjusted mortality rate among racial groups, whereas Asian or Pacific Islander older women had the lowest. Importantly, mortality reflects both the underlying incidence of breast cancer and survival after diagnosis. Therefore, higher mortality among Black older women may reflect a combination of factors, including differences in tumor subtype, stage at diagnosis, comorbidity burden, treatment access, treatment quality, structural inequities, and survivorship care (20, 21). However, these potential mechanisms cannot be disentangled using CDC WONDER death-certificate data, which lack individual-level information on cancer incidence, tumor biology, stage, screening history, treatment receipt, recurrence, comorbidities, frailty, socioeconomic status, and health-system factors. Accordingly, the racial differences observed in this study should be viewed as descriptive mortality disparities rather than evidence of specific causal pathways. Future studies linking mortality records with cancer registry, treatment, comorbidity, and socioeconomic data are needed to clarify the relative contributions of incidence and survival to these disparities.

Sex-stratified analyses showed markedly different mortality burdens between women and men. As expected, women accounted for the overwhelming majority of deaths due to breast cancer and had substantially higher age-adjusted mortality rates than men. However, male breast cancer is a rare disease with distinct epidemiologic, clinical, and biological characteristics, and the male mortality pattern should not be interpreted simply as a low-mortality comparator for female breast cancer (22, 23). In addition, because the number of deaths among men was relatively small compared with women, sex-specific estimates for men may be more sensitive to random variation and should be interpreted cautiously. The observed trends in male breast cancer mortality are therefore best viewed as descriptive surveillance findings rather than evidence of mechanisms shared with female breast cancer. Further studies specifically focused on male breast cancer, with information on stage, tumor subtype, treatment, comorbidities, and health-care access, are needed to better characterize mortality patterns in this population.

The steep age gradient observed in this study highlights the need to interpret breast cancer mortality among older adults through a geriatric oncology framework. Mortality rates were highest among adults aged 85 years or older, a group in whom cancer outcomes are shaped not only by tumor-related factors but also by frailty, multimorbidity, polypharmacy, functional impairment, cognitive status, competing risks of death, social support, and treatment tolerance (24, 25). Therefore, screening and treatment decisions in older patients should be individualized rather than based on chronological age alone. For breast cancer screening, particularly among women older than 75 years, decisions should consider life expectancy, functional status, patient values, and the balance between potential benefits and harms of mammography (26). Similarly, treatment planning should incorporate geriatric assessment to guide surgical candidacy, radiotherapy decisions, endocrine therapy selection and adherence support, chemotherapy selection and dose intensity, and the use of HER2-targeted therapy when clinically appropriate (27). In older patients, endocrine therapy may be influenced by tolerability, bone health, thromboembolic risk, musculoskeletal symptoms, cognition, and adherence; chemotherapy decisions require careful consideration of physiologic reserve, comorbidity burden, toxicity risk, and patient goals; HER2-targeted therapy requires attention to cardiac risk and monitoring; radiotherapy decisions should weigh local-control benefit against treatment burden, life expectancy, and functional limitations; and surgical decisions should consider operative risk, expected benefit, recovery, and preservation of independence. Survivorship care is also particularly important in this population, including management of late treatment effects, cardiotoxicity, bone health, lymphedema, functional decline, cognition, comorbidities, medication burden, and coordination between oncology, primary care, and geriatric services. However, CDC WONDER does not include individual-level information on screening history, tumor stage or subtype, treatment receipt, geriatric assessment, treatment toxicity, recurrence, or survivorship care; thus, the present findings cannot determine how these factors contributed to the observed mortality patterns. Instead, our results underscore the need for future studies linking mortality data with cancer registry, treatment, comorbidity, geriatric assessment, and survivorship data to better define opportunities for improving breast cancer outcomes among older adults.

The SDI analyses provided complementary but distinct perspectives on female state-level mortality. In the pooled jurisdiction-year data, SDI and AAMR showed a modest inverse rank correlation, but this statistic combined between-jurisdiction differences with within-jurisdiction temporal variation and was retained only as an unadjusted descriptive summary. After controlling for jurisdiction and calendar-year fixed effects, within-jurisdiction increases in SDI were associated with higher AAMR. The reversal in direction suggests that the inverse pooled pattern was largely attributable to persistent between-jurisdiction differences and common temporal trends rather than to a negative within-jurisdiction longitudinal association. This result should not be interpreted as evidence that increasing sociodemographic development causes higher mortality, because the panel model remains ecological and may be affected by time-varying confounding, changes in ascertainment, population composition, health-care use, or other unmeasured jurisdiction-level factors. The 2020 descriptive frontier analysis further showed substantial variation among jurisdictions with comparable SDI levels. Smoothed frontier gaps identify jurisdictions warranting further investigation but are not estimates of preventable deaths or the effects of specific interventions.

The present study was restricted to observed mortality data from 1999 to 2020. Accordingly, the findings should be interpreted as a description of historical mortality patterns during the study period.

The observed demographic and geographic variation in breast cancer mortality among older adults has potential implications for public health surveillance and future research, but it should not be interpreted as direct evidence for the effectiveness of specific interventions. In particular, although strategies such as patient navigation, improved access to diagnostic evaluation, geriatric assessment, multidisciplinary treatment coordination, and survivorship support may be relevant to older adults with breast cancer, the present CDC WONDER-based analysis did not directly measure these services or test their impact on mortality. Therefore, our findings are best viewed as identifying populations and geographic areas where further investigation may be warranted. Future studies using linked cancer registry, claims, clinical, geriatric assessment, socioeconomic, and health-system data are needed to determine which modifiable factors contribute to the observed mortality patterns and which interventions are most likely to reduce disparities among older adults.

This study has several strengths. It used a large, nationally representative mortality database covering more than two decades and included comprehensive stratification by demographic and geographic characteristics. The use of Joinpoint regression allowed identification of changes in temporal trends. In addition, the two-way fixed-effects panel model accounted for repeated observations within jurisdictions and national temporal changes, while the two-stage free-disposal-based and LOESS-smoothed frontier procedure provided a reproducible framework for descriptive jurisdiction-level comparison.

Several limitations should be acknowledged. First, death certificate data may be subject to misclassification of the underlying cause of death, and this study included only deaths for which breast cancer was recorded as the underlying cause. Deaths in which breast cancer contributed indirectly may therefore have been missed. Second, CDC WONDER provides aggregate mortality data and does not include individual-level information on tumor stage, receptor subtype, HER2 status, genomic risk, screening history, systemic therapy, endocrine therapy, chemotherapy, HER2-targeted therapy, surgery, radiotherapy, recurrence, treatment adherence, treatment toxicity, frailty, comorbidity, functional status, cognitive status, socioeconomic status, insurance coverage, health-care access, or survivorship care. The database also does not provide information on competing risks or non–breast cancer causes of death at the individual level. Therefore, the present study cannot determine whether observed mortality disparities reflect differences in breast cancer incidence, tumor biology, stage at diagnosis, treatment receipt or quality, survival after diagnosis, comorbidity burden, frailty, socioeconomic context, health-system factors, or competing causes of death. Third, race and ethnicity are recorded on death certificates and may be misclassified, particularly for some minority populations (28). Fourth, some subgroup estimates may be unstable when death counts are small, especially among men and smaller racial groups. Fifth, the SDI and frontier analyses were ecological jurisdiction-level analyses and should not be interpreted as evidence of individual-level associations, causal mechanisms, or the effectiveness of specific interventions.

## Conclusion

In conclusion, breast cancer age-adjusted mortality rates among U.S. adults aged 65 years and older declined substantially from 1999 to 2020, while the absolute number of deaths due to breast cancer increased, reflecting the growing impact of population aging. The study also identified persistent differences in mortality by race, ethnicity, geographic region, state, and urbanization status, indicating that progress has not been evenly distributed across older populations. Because this analysis used aggregate death-certificate data, these differences should be interpreted as descriptive mortality patterns rather than evidence of specific causal mechanisms or direct solutions. Overall, these findings support continued surveillance, further investigation using linked clinical and population-level data, and geriatric-oncology-informed cancer-control strategies that consider individualized screening, competing risks, frailty, treatment tolerance, survivorship needs, and equitable access to high-quality breast cancer care for older adults.

## Data Availability

Publicly available datasets were analyzed in this study. This data can be found here: The mortality data are publicly available on the internet for data users and researchers throughout the world (https://wonder.cdc.gov/).
