# Supplementary material for: Trends and disparities in breast cancer mortality among older adults in the United States, 1999–2020
Source: Front Oncol. 2026 Sep 25;16:1914451. doi: 10.3389/fonc.2026.1914451 (PMC13649381; doi:10.3389/fonc.2026.1914451)
Supplement: Supplementary file 1 [file DataSheet1.docx]

Supplementary Figure 1. Age-specific crude breast cancer mortality rates by sex and age group in 1999 and 2020.

Supplementary Figure 2. Age- and sex-specific annual crude breast cancer mortality rates among adults aged ≥65 years, 1999–2020.

(A) Crude mortality rates by age group in 1999 and 2020; (B) sex-stratified crude mortality rates by age group in 1999 and 2020; (C) annual crude mortality-rate trajectories by age group from 1999 to 2020; (D) annual crude mortality-rate trajectories by sex and age group from 1999 to 2020. All rates are crude rates expressed per 100,000 population and are not age-adjusted.

Supplementary Figure 3. Long-term trends in deaths due to breast cancer, age-adjusted mortality rates, and age distribution, 1999–2020.

(A) Annual number of deaths due to breast cancer and age-adjusted mortality rate (AAMR); (B) annual number of deaths due to breast cancer and AAMR by sex; (C) proportion of annual deaths by age group; (D) proportion of annual deaths by sex and age group. Panels A and B use dual y-axes: bars show the number of deaths on the left y-axis, and lines show AAMR per 100,000 population on the right y-axis. Panels C and D show the proportion (%) of annual deaths by age group; these are proportions of deaths, not mortality rates.

Supplementary Figure 4. Distribution of female breast cancer age-adjusted mortality rates across Socio-Demographic Index levels, 1999–2020.

Points represent jurisdiction-year observations among women aged ≥65 years. Point symbols identify jurisdictions, and point size indicates calendar year. The solid curve summarizes the descriptive nonlinear relationship between the Socio-Demographic Index and female-specific AAMR. AAMR, age-adjusted mortality rate; SDI, Socio-Demographic Index.
